# A wave finite element approach for modelling wave transmission through laminated plate junctions

**DOI:** 10.1038/s41598-022-05685-y

**Published:** 2022-02-03

**Authors:** Nurkanat Aimakov, Gregor Tanner, Dimitrios Chronopoulos

**Affiliations:** 1 School of Mathematical Sciences, University of Nottingham, Nottingham, NG7 2RD UK; 2 Department of Mechanical Engineering and Mecha(tro)nic System Dynamics (LMSD), KU Leuven, 9000 Leuven, Belgium

**Keywords:** Applied mathematics, Composites

## Abstract

We present a numerical method for computing reflection and transmission coefficients at joints connecting composite laminated plates. The method is based on modelling joints with finite elements with boundary conditions given by the solutions of the wave finite element method for the plates in the infinite half-spaces connected to the joint. There are no restrictions on the number of plates, inter-plate angles, and material parameters of individual layers forming the composite. An L-shaped laminated plate junction is discussed in more detail. Comparisons of numerically predicted scattering coefficients with semi-analytical solutions for the selected structures are presented. The results obtained are essential for statistical energy analysis and dynamical energy analysis based calculations of the wave energy distribution in full built-up structure.

## Introduction

Composites are widely used within the transport sector, in particular in the aerospace, automotive and naval manufacturing industries^1,2^. In comparison to isotropic materials such as aluminium and stainless steel, composites provide similar stiffness and strength characteristics whilst being significantly lighter^3^. Furthermore, the mechanical properties of fibre-reinforced composites can be tailored to suit particular needs^1,4^. Over the past decades, these advantages of composites have led to a growing number of use-cases for composites in the construction of primary structural components in the aerospace and automotive industries.

However, despite their superior structural characteristics, composites exhibit reduced vibro-acoustic performance levels due to the large variety of propagating wave modes. Thus, modelling noise and vibration in composite structures plays an important role both at the design phase of a vehicle and at the post-built stage, when non-destructive testing techniques help monitor the structure’s performance. Therefore, there is a need for numerical methods to evaluate the vibrational response of composite structures fast and accurately.

Vibrations of a complex structure are in general modelled using deterministic schemes such as finite element (FE)^5^, finite difference (FD)^6^ or boundary element (BE) methods^7^. These methods are particularly useful in providing the full phase and amplitude information of the wave field in the low-frequency regime. However, at higher frequencies, these methods become inefficient and computationally expensive as the model-size increases drastically with the frequency. Moreover, mode shapes and eigenfrequencies which are essential in the modal approach become highly sensitive to geometrical and/or material uncertainties of meshes, thus producing inaccurate results^8–10^.

At higher frequencies, numerical approaches such as SEA^11,12^, the radiative transfer method^13–15^ or DEA^16–18^ are favoured. For all these methods, wave propagation characteristics such as dispersion relations and scattering coefficients at discontinuities in the structure are required and routinely used. For complex materials such as composites, the dispersion curves and associated mode types can be obtained numerically using the wave finite element (WFE) method. Reflection and transmission behaviour at joints can be solved using FE tools as has been done for isotropic materials, see next paragraphs, and will be presented for composites in “Wave finite element method for composite plates”. Alternatively, these scattering coefficients can be estimated using semi-analytical methods based on force-balance equations at the interface, see^19^ for isotropic materials and^20^ for composite plates.

The WFE method is a technique to study wave motion in homogeneous or periodic structures. The vibro-acoustic behaviour of the whole structure can then be described through the analysis of a single FE-cell or using one periodic segment of the structure, respectively^21^. Since only one period of the structure is used, the size of the WFE model does not depend on the dimensions of the waveguide, and the computational cost of the method is low. In addition, conventional FE matrices are used to discretise the periodic cell; thus, the full potential of existing conventional FE tools can be exploited. The WFE method was originally proposed by Mead^22^ describing the harmonic wave propagation in one-dimensional periodic systems. An important contribution to the analysis of wave propagation in various periodic structures using FE models has been made by Abdel-Rahman^23^. The free wave propagation in one-dimensional isotropic and anisotropic (composite) waveguides was analysed by Mace et al.^24^ and Duhamel et al.^25^. Dispersion relations of waves were computed using the one-dimensional WFE method for structures such as stiffened cylinders^26^, car tyres^27^, thin-walled structures^28^, inhomogeneous cylindrical^29^ and fluid-filled pipes^30^, sandwich beams and panels^31,32^ and laminated cylinders^33^. A detailed analysis of the numerical implementation and numerous applications of the one-dimensional WFE method can be found in the work by Waki^34,35^. It is worth mentioning the work of Mencik and Ichchou^36^ suggesting a special substructuring technique to address numerical issues in the case of multi-layered structures.

The basics of the WFE method for two-dimensional periodic systems were presented in the work by Mead^22^. Later, Mead and Parthan^37^ showed how the problem of defining the dispersion relations in the general direction over the plate’s plane dimensions could be reduced to an array of one-dimensional WFE problems with varying lengths of the periodic segments. A rigorous mathematical framework for the WFE method for two-dimensional periodic isotropic and composite systems has been developed by Manconi and Mace^38,39^. Several representations of the eigenvalue problem leading to the computation of dispersion relations were postulated. Alimonti et al.^40^ extended this work by presenting a contour integral method to compute the non-linear eigenvalue problem arising from the governing equations of motion upon fixing the frequency and the direction of propagation. Dispersion relations were computed for two-dimensional arbitrarily thick layered panels in^41,42^ and periodic textile composites in^43^.

Mencik and Ichchou introduced the hybrid FE/WFE method for calculating reflection and transmission coefficients for one-dimensional waveguides coupled longitudinally^44^. In recent years, this method has been extended to other types of junctions^45–47^ and to two-dimensional waveguides^48,49^. However, the structures considered in the references listed above were all isotropic.

Beyond the case of wave propagation in isotropic materials, Chronopoulos^50^ computed scattering coefficients at a junction representing damage between two composite beams. Later, Apolowo and Chronopoulos^51^ computed the scattering coefficients of two multi-layer composite plates coupled longitudinally to localise the structural damage in the context of structural health monitoring. An attempt to extend the work of Renno et al.^45^ to composite plates has been made by Mitrou and Renno in^52^. The results were not reliable, however, as the energy scattering coefficients did not sum to unity as expected in lossless systems^45,49^.

Beyond the WFE method, Karunasena and Shah^53^ studied reflection of guided waves in the region of a bonding material connecting two composite plates using the hybrid FE and semi-analytical FE method. Bosmans et al.^54^ studied the scattering properties of orthotropic plate junctions with principal material axes aligned with the plate coordinates, that is, so-called *specially orthotropic* plates. Results were presented only for the particular case of bending wave transmission loss in right-angled plates, so-called L-junctions. Aimakov et al.^20^ developed a semi-analytical approach for calculating scattering matrices of junctions of orthotropic plates with no restrictions on the angles of orientation and of the principal material axes. Lee et al.^55^ presented the scattering coefficients of coupled composite plates with joint compliance and damping using the First-Order laminated plate theory^56^. However, as in the work of Bosmans et al., the principal material axes of laminates considered in this work are aligned with the plate coordinates, effectively reducing the complexity of the underlying governing equations. Furthermore, in^55^, a shear correction factor is introduced to correct transverse shear stiffness in the laminate, which must be defined for each laminate separately.

In this paper, we extend the hybrid FE/WFE method to composite laminated plates. The principal novelty of this work is a detailed derivation of reflection and transmission matrices for waves travelling in the structural junctions connecting composite laminated plates at arbitrary angles and with an arbitrary material orientation of the principal axis of the laminae. We give, in particular, the scattering coefficients containing the full angle-of-incidence and frequency dependence. For ray-based methods such as the radiative transfer method and DEA, detailed information on the reflection/transmission behaviour of all propagating modes at complex junctions is needed. This includes information about the angle-of-incidence dependence of scattering and mode conversion coefficients.

The manuscript is organised as follows: in “Wave finite element method for composite plates”, the WFE method for modelling composite plates is reviewed. An eigenvalue problem whose solutions yield wave numbers and mode shapes is set up. The classification of the wave numbers and the wave basis setting are described. Having established a wave basis representation of displacement and force vectors in individual plates, we combine these solutions with the equations of motion of the joint fulfilling continuity of displacements and force equilibrium at the joint boundaries. This then yields the desired scattering coefficients as described in “Hybrid FE/WFE method and scattering coefficients”. In “Numerical case examples”, we present numerical case studies for two coupled composite plates. In particular, the energy scattering coefficients for L-type junctions of regular cross-ply and angle-ply composite plates are computed. These results are compared with semi-analytical estimates of energy scattering coefficients based on the work of Aimakov et al.^20^. Finally, concluding remarks are put in “Conclusion”.

**Figure 1:**
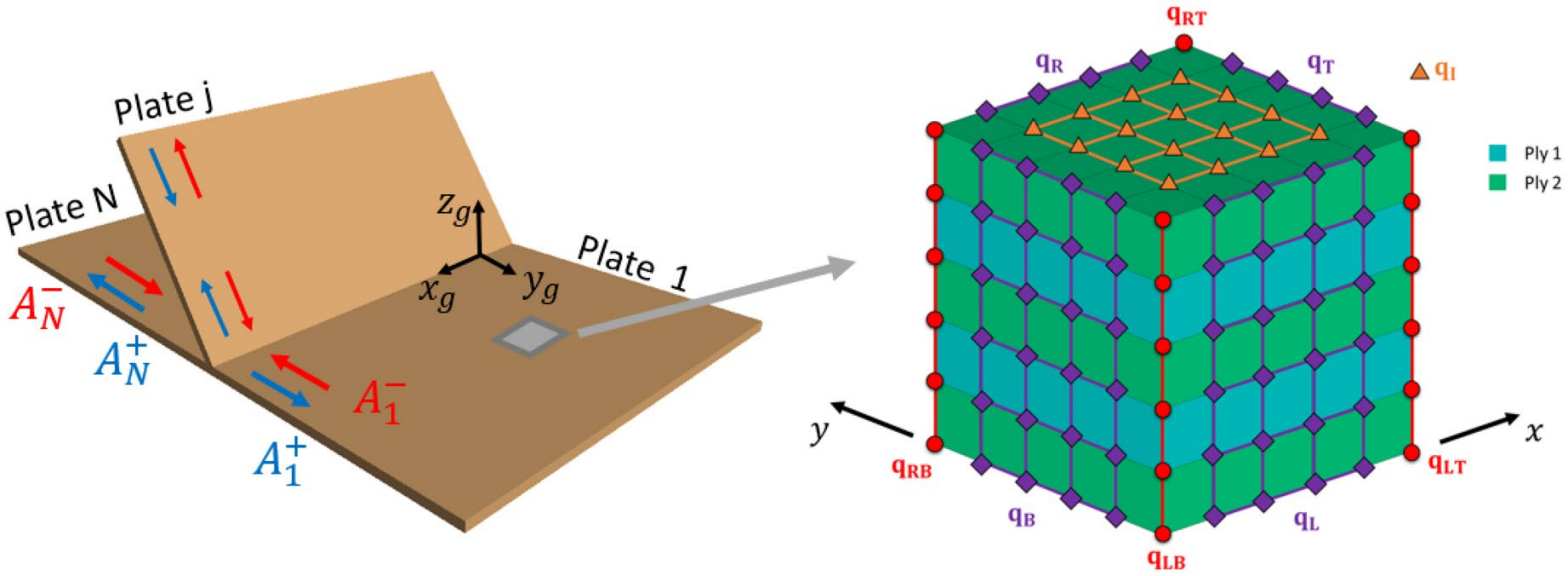
The set up of *N* plates joined together and a periodic segment of a plate with two different alternating plies modelled with three-dimensional finite elements. $A^{\pm }_{1,N}$ are the amplitudes of incoming and outgoing waves travelling from infinity towards the junction and from the junction to infinity, respectively. The degrees of freedom are grouped into internal ${\mathbf {q}}_I$, edge ${\mathbf {q}}_L$, ${\mathbf {q}}_R$, ${\mathbf {q}}_B$, ${\mathbf {q}}_T$ and corner ${\mathbf {q}}_{LB}$, ${\mathbf {q}}_{RB}$, ${\mathbf {q}}_{LT}$, ${\mathbf {q}}_{RT}$ degrees of freedom.

## Wave finite element method for composite plates

### Governing equations of motion

Consider a unit cell of a periodic or homogeneous composite plate with arbitrary lay-up through the thickness direction and plane dimensions $d_x$ and $d_y$. (Note that for a homogeneous structure, the length scales of the unit cell are somewhat arbitrary and typically represented by a single finite element in the plate directions.) It can be modelled using three-dimensional solid elements (such as SOLID185 in the FE software ANSYS) stacked up one on top of the other, representing different composite layers. Figure 1 represents a unit cell of a five-layer plate meshed with SOLID185 elements and a nodal displacements vector labelling convention. The nodal displacements vector ${\mathbf {q}}$ is organised as $${\mathbf {q}} = \textstyle \begin{Bmatrix}{\mathbf {q}}_{LB}&\!{\mathbf {q}}_{RB}&\!{\mathbf {q}}_B&\!{\mathbf {q}}_L&\!{\mathbf {q}}_R&\!{\mathbf {q}}_I&\!{\mathbf {q}}_{LT}&\!{\mathbf {q}}_{RT}&\!{\mathbf {q}}_T\end{Bmatrix}^\mathrm {T}$$. The nodal forces vector ${\mathbf {f}}$ is arranged in the same manner. The number of degrees of freedom must be the same for each pair of edges on opposite faces. The number of mesh cells in the *x*, *y* and *z* direction are labelled by $n_x$, $n_y$ and $n_z$. The number of degrees of freedom per edge is labelled as *m*; for plates modelled with SOLID185 elements $m=3(n_z+1)$. Consequently, the sizes of nodal displacement sub-vectors can be represented as

1 $$\begin{aligned} &|{\mathbf {q}}_{L(R)B(T)}| = m , |{\mathbf {q}}_{L(R)}| = m(n_x-1) ,\\&|{\mathbf {q}}_{B(T)}| = m(n_y-1) , |{\mathbf {q}}_I| = m(n_x-1)(n_y-1) . \end{aligned} $$

Assuming that the structure undergoes harmonic vibration with angular frequency $\omega$ and no external forces are applied, we can write the governing equation of motion of the unit cell as

2 $$\begin{aligned} \left[ {\mathbf {K}}\left( 1+\mathrm {i}\eta \right) -\omega ^2{\mathbf {M}} \right] {\mathbf {q}} = {\mathbf {f}} , \end{aligned}$$

where ${\mathbf {M}}$ and ${\mathbf {K}}$ are the mass and stiffness matrices, respectively. The parameter $\eta$ denotes a uniform structural damping coefficient. The dimension of Eq. (2) is $m(n_x+1)(n_y+1)$.

We consider a plane wave travelling across the plate to be of the form $\exp (-\mathrm {i}k_x x -\mathrm {i}k_y y + \mathrm {i}\omega t)$, where $k_x$ and $k_y$ are the *x* and *y* components of the wave vector ${\mathbf {k}}$. Periodic structure theory^57,58^ requires that

3 $$\begin{aligned} \begin{Bmatrix} {\mathbf {q}}_{LT} \\ {\mathbf {q}}_{RT} \\ {\mathbf {q}}_T \end{Bmatrix} = \lambda _x \begin{Bmatrix} {\mathbf {q}}_{LB} \\ {\mathbf {q}}_{RB} \\ {\mathbf {q}}_B \end{Bmatrix} , \end{aligned}$$

where $\lambda _x = e^{-\mathrm {i}k_x d_x}$ is the propagation factor in the *x* direction. Now, we denote as ${\mathbf {q}}^j_{X}$, $X = L, R, I$ displacement sub-vectors of nodes placed at $x_j = d_x j /n_x$, $j = 1,2,\dots ,n_x-1$. Consequently, as in Eq. (3), one can relate internal and edge degrees of freedom to bottom ones as

4 $$\begin{aligned} \begin{Bmatrix} {\mathbf {q}}^j_L \\ {\mathbf {q}}^j_R \\ {\mathbf {q}}^j_I \end{Bmatrix} = e^{-\mathrm {i}k_x d_x j /n_x} \begin{Bmatrix} {\mathbf {q}}_{LB} \\ {\mathbf {q}}_{RB} \\ {\mathbf {q}}_B \end{Bmatrix} = \lambda ^{j/n_x}_x \begin{Bmatrix} {\mathbf {q}}_{LB} \\ {\mathbf {q}}_{RB} \\ {\mathbf {q}}_B \end{Bmatrix}. \end{aligned}$$

Following Eqs. (3) and (4), we can express the nodal displacements vector ${\mathbf {q}}$ in terms of displacement sub-vectors of nodes $${\mathbf {q}}_{red} = \textstyle \begin{Bmatrix}{\mathbf {q}}_{LB}&\!{\mathbf {q}}_{RB}&\!{\mathbf {q}}_B\end{Bmatrix}^\mathrm {T} \equiv \textstyle \begin{Bmatrix}{\tilde{\mathbf{q}}}_L&\!{\tilde{\mathbf{q}}}_R&\!{\tilde{\mathbf{q}}}_O\end{Bmatrix}^\mathrm {T}$$ as

5 $$\begin{aligned} {\mathbf {q}} = {\mathbf {T}} {\mathbf {q}}_{red} , \quad {\mathbf {T}} = \begin{bmatrix} \mathbf {I_1} &{} 0 &{} 0 \\ 0 &{} \mathbf {I_1} &{} 0 \\ 0 &{} 0 &{} \mathbf {I_2} \\ \vdots \\ \lambda ^{j/n_x}_x\,\mathbf {I_1} &{} 0 &{} 0 \\ 0 &{} \lambda ^{j/n_x}_x\,\mathbf {I_1} &{} 0 \\ 0 &{} 0 &{} \lambda ^{j/n_x}_x\,\mathbf {I_2} \\ \vdots \\ \lambda _x\,\mathbf {I_1} &{} 0 &{} 0 \\ 0 &{} \lambda _x\,\mathbf {I_1} &{} 0 \\ 0 &{} 0 &{} \lambda _x\,\mathbf {I_2} \end{bmatrix} ,\quad j = 1,2,\dots ,n_x-1 , \end{aligned}$$

where ${\mathbf {I}}_1$ and ${\mathbf {I}}_2$ are identity matrices with dimensions *m* and $m(n_y-1)$. The dimension of the matrix ${\mathbf {T}}$ is $\left( m(n_x+1)(n_y+1),\,m(n_y+1)\right)$, and hence, Eq. (5) can be used to reduce the dimension of Eq. (2) as follows

6 $$\begin{aligned} {\mathbf {D}}{\mathbf {q}}_{red} = {\mathbf {f}}_{red} , \quad {\mathbf {D}} = {\mathbf {T}}^H \left[ {\mathbf {K}}\left( 1+\mathrm {i}\eta \right) -\omega ^2{\mathbf {M}}\right] {\mathbf {T}} , \end{aligned}$$

and

7 $$\begin{aligned} {\mathbf {f}}_{red} = \begin{Bmatrix} {\tilde{\mathbf{f}}}_L \\ {\tilde{\mathbf{f}}}_R \\ {\tilde{\mathbf{f}}}_O \end{Bmatrix} = {\mathbf {T}}^H\, {\mathbf {f}} = \begin{Bmatrix} {\mathbf {f}}_{LB} + \displaystyle \sum _{j=1}^{n_x-1} \lambda ^{-j/n_x}_x\, {\mathbf {f}}^j_L + \lambda ^{-1}_x {\mathbf {f}}_{LT}\\ {\mathbf {f}}_{RB} + \displaystyle \sum _{j=1}^{n_x-1} \lambda ^{-j/n_x}_x\, {\mathbf {f}}^j_R + \lambda ^{-1}_x {\mathbf {f}}_{RT} \\ {\mathbf {f}}_{B} + \displaystyle \sum _{j=1}^{n_x-1} \lambda ^{-j/n_x}_x\, {\mathbf {f}}^j_I + \lambda ^{-1}_x {\mathbf {f}}_{T} \end{Bmatrix} , \end{aligned}$$

where ${\mathbf {f}}^j_{X}$, $X = L, R, I$ denote force sub-vectors of nodes placed at $x_j = d_x j /n_x$, $j = 1,2,\dots ,n_x-1$. ${\mathbf {D}}$ is the reduced dynamic stiffness matrix with dimensions $\left( m(n_y+1),m(n_y+1)\right)$. Internal nodal forces ${\mathbf {f}}^j_{I} = 0$ in the absence of external forces, and force equilibrium between opposite sides yields $\lambda _x{\mathbf {f}}_B + {\mathbf {f}}_T = 0$. Therefore, the reduced nodal forces vector ${\tilde{\mathbf{f}}}_O = 0$, thus allowing to reduce further the dimension of Eq. (6) via dynamic condensation of internal degrees of freedom ${\tilde{\mathbf{q}}}_O$^35^:

8 $$\begin{aligned} \begin{bmatrix} {\tilde{\mathbf{D}}}_{LL} &{} {\tilde{\mathbf{D}}}_{LR} \\ {\tilde{\mathbf{D}}}_{RL} &{} {\tilde{\mathbf{D}}}_{RR} \end{bmatrix} \begin{Bmatrix} {\tilde{\mathbf{q}}}_L \\ {\tilde{\mathbf{q}}}_R \end{Bmatrix} = \begin{Bmatrix} {\tilde{\mathbf{f}}}_L \\ {\tilde{\mathbf{f}}}_R \end{Bmatrix} , \end{aligned}$$

where

9 $$\begin{aligned} &{\tilde{\mathbf{D}}}_{LL} = {\mathbf {D}}_{LL} - {\mathbf {D}}_{LO} {\mathbf {D}}_{OO}^{-1} {\mathbf {D}}_{OL} \\&{\tilde{\mathbf{D}}}_{LR} = {\mathbf {D}}_{LR} - {\mathbf {D}}_{RO} {\mathbf {D}}_{OO}^{-1} {\mathbf {D}}_{OR} \\&{\tilde{\mathbf{D}}}_{RL} = {\mathbf {D}}_{RL} - {\mathbf {D}}_{LO} {\mathbf {D}}_{OO}^{-1} {\mathbf {D}}_{OL} \\&{\tilde{\mathbf{D}}}_{RR} = {\mathbf {D}}_{RR} - {\mathbf {D}}_{RO} {\mathbf {D}}_{OO}^{-1} {\mathbf {D}}_{OR} \end{aligned} . $$

Finally, by applying the periodic structure theory and force equilibrium in the *y* direction, which can be written as

10 $$\begin{aligned} {\tilde{\mathbf{q}}}_R = \lambda _y\,{\tilde{\mathbf{q}}}_L , \quad {\tilde{\mathbf{f}}}_R = -\lambda _y\,{\tilde{\mathbf{f}}}_L , \quad \lambda _y = e^{-\mathrm {i}k_y d_y} , \end{aligned}$$

one can get from Eq. (8) the following eigenvalue problem for the propagation factor $\lambda _y$

11 $$\begin{aligned} {\mathbf {Z}} \begin{Bmatrix} {\tilde{\mathbf{q}}}_L \\ {\tilde{\mathbf{f}}}_L \end{Bmatrix} = \lambda _y \, \begin{Bmatrix} {\tilde{\mathbf{q}}}_L \\ {\tilde{\mathbf{f}}}_L \end{Bmatrix} \quad \text {with} \quad {\mathbf {Z}} = \begin{bmatrix} -{\tilde{\mathbf{D}}}_{LR}^{-1}\, {\tilde{\mathbf{D}}}_{LL} &{} {\tilde{\mathbf{D}}}_{LR}^{-1} \\ -{\tilde{\mathbf{D}}}_{RL} + {\tilde{\mathbf{D}}}_{RR}\, {\tilde{\mathbf{D}}}_{LR}^{-1} \, {\tilde{\mathbf{D}}}_{LL} &{} 
-{\tilde{\mathbf{D}}}_{RR}\, {\tilde{\mathbf{D}}}_{LR}^{-1} \end{bmatrix} . \end{aligned}$$

The dimension of the matrices ${\tilde{\mathbf{D}}}$ and ${\mathbf {Z}}$ is 2*m*. Therefore, the solution of the eigenvalue problem (11) consists of 2*m* propagation factors $\lambda _{y,i}$ and the correspondent eigenvectors $$\begin{Bmatrix} \phi _{q,i}&\! \phi _{f,i} \end{Bmatrix}^T$$ provided that the circular frequency $\omega$ and the wave number component $k_x$ are fixed. The wave number components $k_{y,i}$ can be computed as

12 $$\begin{aligned} k_{y,i} = \ln \left( \frac{\lambda _{y,i}}{-\mathrm {i}d_y}\right) , \quad i = 1,\ldots ,2m . \end{aligned}$$

Consequently, by varying the wave number component $k_x$, one can extract wave vector curves $(k_x,k_y)$ for a fixed value of $\omega$. It is worth noting that the so obtained wave number components $k_{y,i}$ can be real, imaginary or complex, making the correspondent plane waves propagating, evanescent or attenuating.

**Table 1:** Engineering constants of Epoxy Carbon UD (230 GPa) material used for individual laminas of a composite plate.

$E_1 \,(\text {N}/\text {m}^2)$	$E_2 \,(\text {N}/\text {m}^2)$	$E_3 \,(\text {N}/\text {m}^2)$	$G_{12} \,(\text {N}/\text {m}^2)$	$G_{23} \,(\text {N}/\text {m}^2)$	$G_{13} \,(\text {N}/\text {m}^2)$
$121 \times 10^9$	$8.6 \times 10^9$	$8.6 \times 10^9$	$4.7 \times 10^9$	$3.1 \times 10^9$	$4.7 \times 10^9$
$\nu _{12}$	$\nu _{23}$	$\nu _{13}$	$\rho \,(\text {kg}/\text {m}^3)$		
0.27	0.4	0.27	1490		

### Computation of wave numbers and eigenvectors

As mentioned in^35^, the solution of the eigenvalue problem (11) might be prone to ill-conditioning of the eigenvalue matrix ${\mathbf {Z}}$ and eigenvectors $[{\tilde{\mathbf{q}}}^j_L , {\tilde{\mathbf{f}}}^j_L]^T$. An equivalent form of the eigenvalue problem can be formulated by eliminating ${\tilde{\mathbf{f}}}_L$ and ${\tilde{\mathbf{f}}}_R$ in Eq. (8) as suggested in^59^:

13 $$\begin{aligned} {\mathbf {N}} \begin{Bmatrix} {\tilde{\mathbf{q}}}_L \\ {\tilde{\mathbf{q}}}_R \end{Bmatrix} = \lambda _y {\mathbf {L}} \begin{Bmatrix} {\tilde{\mathbf{q}}}_L \\ {\tilde{\mathbf{q}}}_R \end{Bmatrix} , \quad {\mathbf {N}} = \begin{bmatrix} 0 &{} {\mathbf {I}} \\ -{\tilde{\mathbf{D}}}_{RL} &{} -{\tilde{\mathbf{D}}}_{RR} \end{bmatrix} \quad , \quad {\mathbf {L}} = \begin{bmatrix} {\mathbf {I}} &{} 0 \\ {\tilde{\mathbf{D}}}_{LL} &{} {\tilde{\mathbf{D}}}_{LR} \end{bmatrix} , \end{aligned}$$

with ${\mathbf {I}}$ being the *m*-by-*m* identity matrix. The matrices ${\mathbf {N}}$ and ${\mathbf {L}}$ consist of block parts of the reduced dynamic stiffness matrix ${\tilde{\mathbf{D}}}$ with no matrix inversion as in Eq. (11), thus reducing numerical ill-conditioning of the method to some extent. However, there might be a difference of several orders of magnitude between ${\mathbf {I}}$ and $-{\tilde{\mathbf{D}}}_{RL(RR)}$ and ${\tilde{\mathbf{D}}}_{LL(LR)}$; therefore, the condition numbers of the matrices ${\mathbf {N}}$ and ${\mathbf {L}}$ can still be large, causing numerical errors in the evaluation of eigenvalues and eigenvectors.

We use the following form of the eigenvalue problem (13), proposed by Fan et al.^47,60^

14 $$\begin{aligned} {\tilde{\mathbf{N}}} \begin{Bmatrix} {\tilde{\mathbf{q}}}_L \\ {\tilde{\mathbf{q}}}_R \end{Bmatrix} = \lambda _y {\tilde{\mathbf{L}}} \begin{Bmatrix} {\tilde{\mathbf{q}}}_L \\ {\tilde{\mathbf{q}}}_R \end{Bmatrix} , \quad {\tilde{\mathbf{N}}} = \begin{bmatrix} 0 &{} \sigma {\mathbf {I}} \\ -{\tilde{\mathbf{D}}}_{RL} &{} -{\tilde{\mathbf{D}}}_{RR} \end{bmatrix} \quad , \quad {\tilde{\mathbf{L}}} = \begin{bmatrix} \sigma {\mathbf {I}} &{} 0 \\ {\tilde{\mathbf{D}}}_{LL} &{} {\tilde{\mathbf{D}}}_{LR} \end{bmatrix} , \end{aligned}$$

where $\sigma = \dfrac{\Vert {\tilde{\mathbf{D}}}_{RR}\Vert _2}{m^2}$, $\Vert \Vert _2$ representing the largest singular value of a matrix. This formulation is an improved version of the eigenvalue problem (13), and the factor $\sigma$ is introduced here to reduce the condition number of the matrices ${\mathbf {N}}$ and ${\mathbf {L}}$. All the formulations presented are equivalent, and the eigenvalue solutions are the same. In fact, writing the characteristic equation of the eigenvalue problem (14) yields

15 $$\begin{aligned} 0 = \det ({\tilde{\mathbf{N}}}-\lambda _y {\tilde{\mathbf{L}}}) = \sigma ^m \det ({\mathbf {N}}-\lambda _y {\mathbf {L}}) . \end{aligned}$$

Note that eigenvectors in Eq. (14) consist of left and right nodal displacements sub-vectors therefore to get an eigenvector in the form as in Eq. (11) one can apply the following transformation

16 $$\begin{aligned} \begin{Bmatrix} {\tilde{\mathbf{q}}}_L \\ {\tilde{\mathbf{f}}}_L \end{Bmatrix} = \begin{bmatrix} {\mathbf {I}} &{} 0 \\ {\tilde{\mathbf{D}}}_{LL} &{} {\tilde{\mathbf{D}}}_{LR} \end{bmatrix} \begin{Bmatrix} {\tilde{\mathbf{q}}}_L \\ {\tilde{\mathbf{q}}}_R \end{Bmatrix} . \end{aligned}$$

### Incoming and outgoing waves and the wave basis

**Figure 2:**
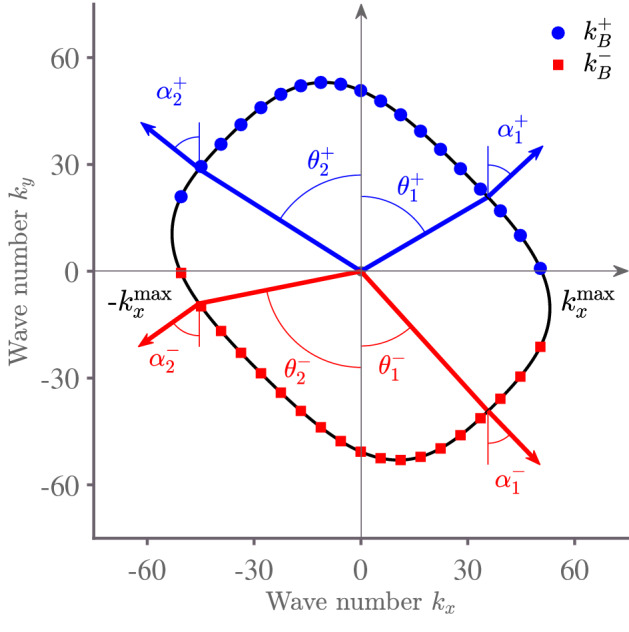
Bending wave vector curve in a $45^{\circ }/-45^{\circ }/45^{\circ }/-45^{\circ }/45^{\circ }$ composite plate at a frequency 3000 Hz. Blue dots represent wave numbers related to outgoing waves while red squares correspond to incoming waves. Angles $\alpha _{1(2)}$ and $\theta _{1(2)}$ represent group and wave vector or phase angles, respectively. The wave number component $k^{\mathrm{max}}_x$ is equal to $53\, \mathrm{m}^{-1}$. Material parameters of orthotropic layers are from Table 1.

**Table 2:** Properties of eigenvalues and associated waves. The expression ${\text {Re}}(\mathrm {i}\omega \phi ^*_{q,i}\, \phi _{f,i})$ denotes the power flow of the *i*th wave.

	Incoming	Outgoing
Propagating $\Leftrightarrow {\text {Re}}(k_{y,i}) > c \,{\text {Im}}(k_{y,i})$	${\text {Re}}(\mathrm {i}\omega \phi ^*_{q,i}\, \phi _{f,i}) < 0$	${\text {Re}}(\mathrm {i}\omega \phi ^*_{q,i}\, \phi _{f,i}) > 0$
Attenuating $\,\Leftrightarrow {\text {Re}}(k_{y,i}) \le c \,{\text {Im}}(k_{y,i})$	${\text {Im}}(k_{y,i}) > 0$	${\text {Im}}(k_{y,i}) < 0$

The eigensolutions of Eq. (14) can be separated into *m* pairs of roots, $\lambda ^\pm _{y,i}$; these correspond to negative waves (with superscripts “−”) and positive waves (with superscripts “$+$”). In the context of scattering properties at junctions, the negative and positive waves will also refer to incoming and outgoing waves moving towards or away from the junction, respectively, see the left-hand-side of Fig. 1. Furthermore, the waves can be categorised as propagating, evanescent or attenuating.

In the absence of damping, the standard wave classification^24,49,61^ consists of checking whether $|\lambda _{y,i}| = 1$ or not to identify propagating or evanescent waves, respectively. If the damping parameter is not zero, we establish an algorithm for the *i*th wave as shown in Table 2, where ${\text {Re}}(\mathrm {i}\omega \phi ^*_{q,i}\, \phi _{f,i})$ is the energy flux of the *i*th wave in the positive *y* direction. The real parameter *c* in Table 2 is chosen empirically to separate the least attenuating waves from evanescent or strongly attenuating waves.

In the case of isotropic materials and some special types of composite plates, e.g. cross-ply laminates, which consist of layers with ply direction angles $0^{\circ }$ or $90^{\circ }$, the transfer matrix ${\mathbf {Z}}$ in Eq. (11) is symplectic. That means that the eigenvalues of Eqs. (11) and (14) appear in pairs $(\lambda _{y,i},1/\lambda _{y,i})$, and $k^+_{y,i} = -k^-_{y,i}$. However, for general composites, the transfer matrix ${\mathbf {Z}}$ is not symplectic, and $k^+_{y,i} \ne -k^-_{y,i}$. Figure 2 shows an example of a bending wave vector curve, where one can see the inequality between incoming and outgoing wave number components $k_y$ represented by red squares and blue dots, respectively. Furthermore, we note the difference between phase and group angles in composite plates shown with $\theta ^\pm _{1,2}$ and $\alpha ^\pm _{1,2}$, respectively. These features were discussed for a similar wave vector curve in^20,62^.

**Figure 3:**
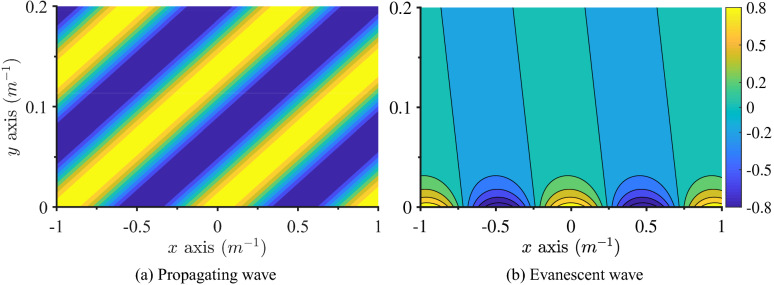
Contour plot of the real part of the out-of-plane displacement produced by the outgoing propagating (**a**) and evanescent (**b**) bending waves in a $45^{\circ }/-45^{\circ }/45^{\circ }/-45^{\circ }/45^{\circ }$ composite plate plotted in the (*x*, *y*) plane. Brighter colours represent positive *z* values, whilst darker ones denote negative *z* values.

Recall that in the absence of damping, we expect that waves are either propagating or evanescent. While the wave number components $k^{\pm }_{y,i}$ for propagating waves are indeed purely real, those of non-propagating waves can still have both a real and imaginary part for composites, thus seemingly producing an energy flux in the *y* direction^20^. However, it turns out that these waves are actually purely evanescent and decay or increase exponentially towards the line $y=0$, say, however, with the decaying/increasing direction axis not necessarily aligned with the *y* axis, see^63,64^.

Figure 3 presents the contour plot of the real part of the out-of-plane displacement field created by the outgoing bending propagating (a) and evanescent (b) waves in a $45^{\circ }/-45^{\circ }/45^{\circ }/-45^{\circ }/45^{\circ }$ composite plate. In the case of the evanescent wave, the displacement at $y=0$ along the *x* axis is oscillating, as expected. In contrast, at $y>0$, it decays away along the inclined null-lines, i.e., lines at which displacement is zero (black straight lines in Fig. 3b). This leads to the oscillating displacement shape projection along the *y* axis, which is why the energy flux along the *y* axis appears to be non-zero.

**Figure 4:**
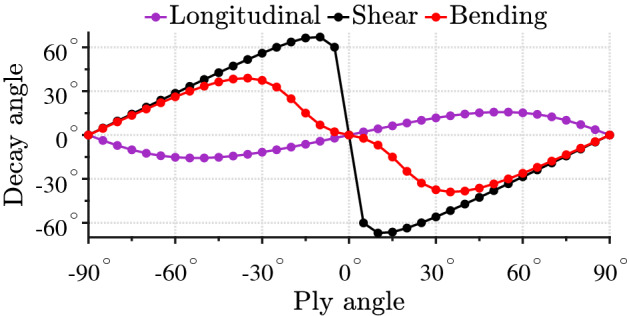
Decay direction angles of various evanescent waves as a function of a ply direction angle in an orthotropic plate with material parameters from Table 1.

The angle of decay/increase of the evanescent waves depends on the angle of orientation of the principal material axes of plies of the plate, i.e. on the ply direction angle. It can be computed as $\arctan ({\text {Re}}(k^\pm _{y,i})/k_x)$. Figure 4 presents the decay direction angles of longitudinal, shear and bending evanescent waves with respect to the *y* axis as a function of the ply direction angle of an orthotropic plate with material parameters from Table 1. It can be noted that the decay of evanescent waves can be inclined with respect to the *y* axis with angles up to $60^{\circ }$; for example, the line corresponding to the evanescent shear wave in Fig. 4. We can see that the decay angles are zero for ply direction of angles $0^{\circ }$ and $\pm 90^{\circ }$, that is, the evanescent waves decay/increase along the *y* axis. In such cases, the plate is *specially orthotropic* if it consists of only one layer^20^ or balanced if it consists of multiple layers^1,56^.

Identifying correctly whether a wave is incoming/outgoing and propagating/attenuating is not enough to further proceed with calculating scattering coefficients. In fact, for a range of frequencies and wave number components $k_x$, we obtain a set of unclassified branches of propagating, evanescent and attenuating waves. To identify the eigensolutions of Eq. (14) corresponding to the same wave type, we apply the so-called MAC criterion^65^. For an eigenvector solution $$\Phi _i = \begin{Bmatrix} \phi ^{\pm }_{q,i}&\! \phi ^{\pm }_{f,i} \end{Bmatrix}^T$$ defined at frequency $\omega$ for a fixed wave number component $k_x$, we find an eigenvector solution $$\Phi _j = \begin{Bmatrix} \phi ^{\pm }_{q,j}&\! \phi ^{\pm }_{f,j} \end{Bmatrix}^T$$ at the frequency $\omega +\text {d}\omega$ with sufficiently small d$\omega$ such that

17 $$\begin{aligned} M^{\pm }(\omega ) = \frac{\left( \Phi _i^T(\omega ) \Phi _j^*(\omega +\text {d}\omega )\right) \left( \Phi _j^T(\omega ) \Phi _i^*(\omega +\text {d}\omega )\right) }{\left( \Phi _i^T(\omega ) \Phi _i^*(\omega +\text {d}\omega )\right) \left( \Phi _j^T(\omega ) \Phi _j^*(\omega +\text {d}\omega )\right) } \end{aligned}$$

is maximised. The same criterion can be utilised to classify wave types for a range of wave number components $k_x$ and a fixed frequency with corresponding step size d$k_x$ being sufficiently small.

Once the appropriate pairs of wave number components $k^\pm _{y,i}$ and wave mode shapes $$\begin{Bmatrix} \phi ^\pm _{q,i}&\! \phi ^\pm _{f,i} \end{Bmatrix}^T$$ are determined, we can express nodal displacements and forces in the basis of wave mode shapes as follows

18 $$\begin{aligned} \begin{Bmatrix} {\tilde{\mathbf{q}}}_L \\ {\tilde{\mathbf{f}}}_L \end{Bmatrix} = \sum _{i=1}^m\left( a^+_i \begin{Bmatrix} \phi ^+_{q,i} \\ \phi ^+_{f,i} \end{Bmatrix} + a^-_i \begin{Bmatrix} \phi ^-_{q,i} \\ \phi ^-_{f,i} \end{Bmatrix}\right) = \begin{Bmatrix} \mathbf {\Phi ^+_q}{\mathbf {a}}^+ + \mathbf {\Phi ^-_q}{\mathbf {a}}^- \\ \mathbf {\Phi ^+_f}{\mathbf {a}}^+ + \mathbf {\Phi ^-_f}{\mathbf {a}}^- \end{Bmatrix} , \end{aligned}$$

where $a^+_i$ and $a^-_i$ are the amplitudes of the *i*th outgoing and incoming waves.

## Hybrid FE/WFE method and scattering coefficients

In this section, we derive the energy scattering coefficients using continuity and equilibrium conditions at the boundaries of the joint.

**Figure 5:**
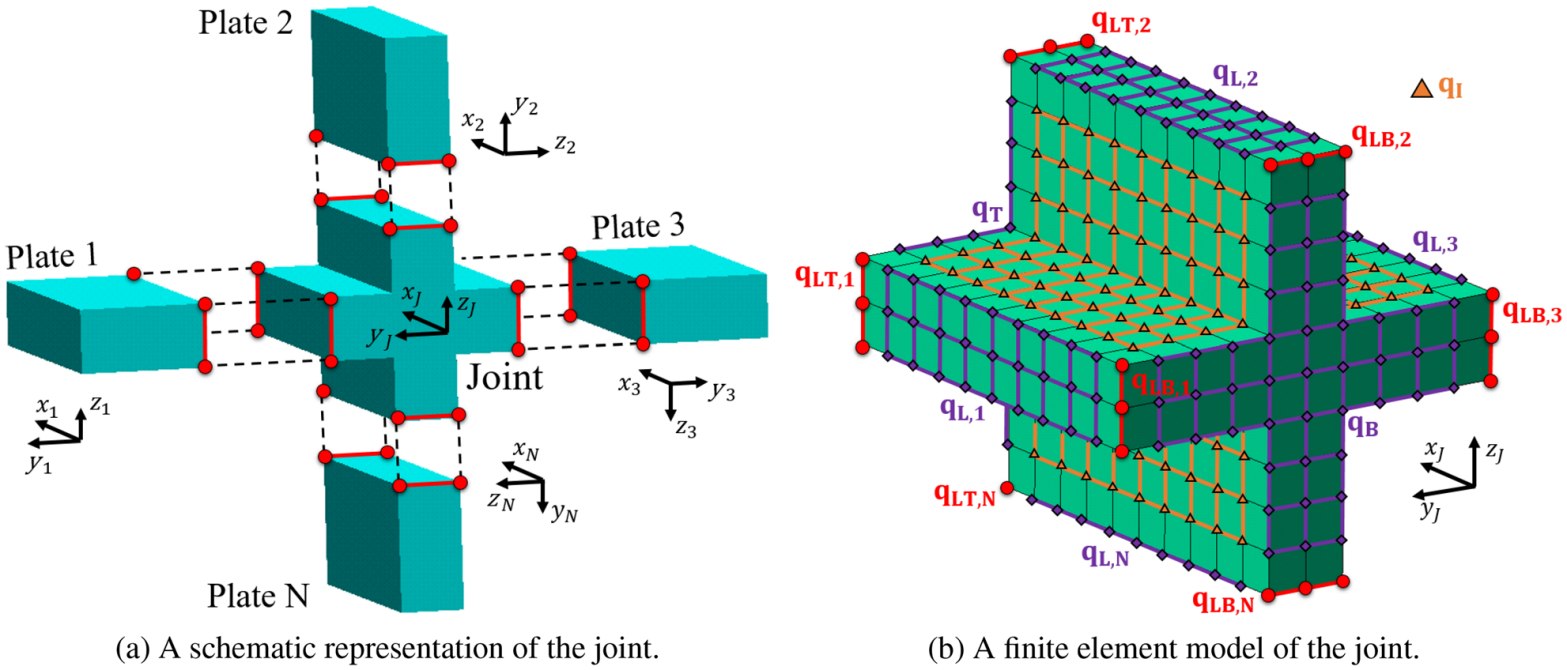
A schematic representation of the joint connecting *N* plates (**a**) and its finite element model (**b**). The *x* axis of all plates are aligned with the $x_J$ axis of the joint. The degrees of freedom are grouped into internal ${\mathbf {q}}_I$, edge ${\mathbf {q}}_B, {\mathbf {q}}_T, {\mathbf {q}}_{L,1},\dots ,{\mathbf {q}}_{L,N}$ and corner ${\mathbf {q}}_{LB,1},\dots ,{\mathbf {q}}_{LB,N},{\mathbf {q}}_{LT,1},\dots ,{\mathbf {q}}_{LT,N}$ degrees of freedom.

### Governing equations of the joint

We consider *N* composite plates that are connected together via a joint element, see Fig. 5a. The local *y* axes of all plates are directed away from the joint, whereas the *x* axes are aligned with the $x_J$ axis of the joint. The joint is assumed to be periodic in the $x_J$ axis, and the width of the periodic segment is assumed to be equal to $d_y$. According to Fig. 5b, the nodal displacements vector of the joint is organised in the following way

19 $$\begin{aligned} {\mathbf {q}}_J = \begin{Bmatrix} {\mathbf {q}}_E&{\mathbf {q}}_O \end{Bmatrix}^\mathrm {T} , \begin{aligned} {\mathbf {q}}_E&= \begin{Bmatrix} {\mathbf {q}}_{LB,1} & {\mathbf {q}}_{L,1} & {\mathbf {q}}_{LT,1} & \ldots & {\mathbf {q}}_{LB,N} & {\mathbf {q}}_{L,N} & {\mathbf {q}}_{LT,N} \end{Bmatrix} \\ {\mathbf {q}}_O&= \begin{Bmatrix} {\mathbf {q}}_B & {\mathbf {q}}_I & {\mathbf {q}}_T \end{Bmatrix} \end{aligned} . \end{aligned}$$

The nodal displacement vector ${\mathbf {q}}_O$ consists of degrees of freedom of the internal nodes. It is required that the node arrangement on the face containing ${\mathbf {q}}_{LB,k}$, ${\mathbf {q}}_{L,k}$ and ${\mathbf {q}}_{LT,k},\,k=1,\ldots ,N$ is coherent with one on the left face of the *k*th plate. Therefore, we enforce that $|{\mathbf {q}}_{LB,k}|=|{\mathbf {q}}_{L,k}|=|{\mathbf {q}}_{LT,k}|=|{\tilde{\mathbf{q}}}_{L,k}|=m_k$ and $n_{J,k}=n_{x,k}$, where $n_{J,k}$ is the number of mesh cells in the joint element along the *x* direction of the face containing ${\mathbf {q}}_{LB,k}$, ${\mathbf {q}}_{L,k}$ and ${\mathbf {q}}_{LT,k}$. The nodal forces vector ${\mathbf {f}}_J$ is arranged in the same way.

The governing equations of motion of the joint are of the same form as in Eq. (2), that is,

20 $$\begin{aligned} {\mathbf {D}}_J {\mathbf {q}}_J = {\mathbf {f}}_J , \quad {\mathbf {D}}_J = {\mathbf {K}}_J(1+\mathrm {i}\eta )-\omega ^2{\mathbf {M}}_J , \end{aligned}$$

where ${\mathbf {K}}_J$ and ${\mathbf {M}}_J$ are the stiffness and mass matrices of the joint. When no external forces are applied on the internal nodes of the joint, Eq. (20) can be written as

21 $$\begin{aligned} \begin{bmatrix} {\mathbf {D}}_{EE} &{} {\mathbf {D}}_{EI} \\ {\mathbf {D}}_{IE} &{} {\mathbf {D}}_{II} \end{bmatrix}\, \begin{Bmatrix} {\mathbf {q}}_E \\ {\mathbf {q}}_O \end{Bmatrix} = \begin{Bmatrix} {\mathbf {f}}_E \\ 0 \end{Bmatrix} , \end{aligned}$$

where ${\mathbf {f}}_O=0$. Consequently, one can remove internal degrees of freedom ${\tilde{\mathbf{q}}}_I$ using the dynamic condensation as

22 $$\begin{aligned} {\mathbf {D}}_{J,\text {cond}}\,{\mathbf {q}}_E = {\mathbf {f}}_E ,\quad {\mathbf {D}}_{J,\text {cond}} = {\mathbf {D}}_{EE} -{\mathbf {D}}_{EI}{\mathbf {D}}_{II}^{-1}{\mathbf {D}}_{IE} . \end{aligned}$$

Now, following a similar approach as in “Governing equations of motion”, we apply periodic structure theory on the nodal displacement sub-vectors along the $x_J$ axis and obtain the following equation

23 $$\begin{aligned} {\tilde{\mathbf{D}}}_J{\mathbf {q}}_{J,red} = {\mathbf {f}}_{J,red} , \quad {\tilde{\mathbf{D}}}_J = {\mathbf {T}}^*_J{\mathbf {D}}_{J,\text {cond}}{\mathbf {T}}_J \end{aligned}$$

with

24 $$\begin{aligned} {\mathbf {f}}_{J,red} = \begin{Bmatrix} {\tilde{\mathbf{f}}}_{LB,1} \\ \vdots \\ {\tilde{\mathbf{f}}}_{LB,N} \end{Bmatrix} = {\mathbf {T}}^*_J\, {\mathbf {f}}_J = \begin{Bmatrix} {\mathbf {f}}_{LB,1} + \displaystyle \sum _{j_1=1}^{n_{J,1}-1} \lambda ^{-j_1/n_{J,1}}_x\, {\mathbf {f}}^{j_1}_{L,1} + \lambda ^{-1}_x {\mathbf {f}}_{LT,1}\\ \vdots \\ {\mathbf {f}}_{LB,N} + \displaystyle \sum _{j_N=1}^{n_{J,N}-1} \lambda ^{-j_N/n_{J,N}}_x\, {\mathbf {f}}^{j_N}_{L,N} + \lambda ^{-1}_x {\mathbf {f}}_{LT,N} \end{Bmatrix} \end{aligned}$$

and

25 $$\begin{aligned} &{\mathbf {q}}_{J,red} = {\mathbf {T}}_J {\mathbf {q}}_E , \quad {\mathbf {q}}_{J,red} = \begin{Bmatrix} {\mathbf {q}}_{LB,1}&\cdots&{\mathbf {q}}_{LB,N} \end{Bmatrix}^{\mathrm {T}} \\&{\mathbf {T}}_J = \begin{bmatrix} {\mathbf {I}}_1 &{} \cdots &{} 0 \\ \vdots &{} \vdots &{} \vdots \\ \lambda ^{j_1/n_{J,1}}_x\,{\mathbf {I}}_1 &{} \cdots &{} 0 \\ \vdots &{} \ddots &{} \vdots \\ 0 &{} \cdots &{} {\mathbf {I}}_N \\ \vdots &{} \vdots &{} \vdots \\ 0 &{} \cdots &{} \lambda ^{j_N/n_{J,N}}_x\,{\mathbf {I}}_N \\ \vdots &{} \vdots &{} \vdots \\ 0 &{} \cdots &{} \lambda _x\,{\mathbf {I}}_N \\ \end{bmatrix} \end{aligned} ,$$

where ${\mathbf {I}}_k$ is the $m_k$-by-$m_k$ identity matrix and $j_k=1,\ldots ,n_{J,k}-1,k=1,\ldots ,N$.

### Reflection and transmission at interfaces of the joint

Equation (23) considers only interface nodes, that is, nodes shared between the plates and the joint. The force equilibrium and displacement continuity conditions must be satisfied at these nodes. Specifically, ${\mathbf {q}}_{LB,k}$ and ${\tilde{\mathbf{f}}}_{LB,k},~k=1,\dots ,N$ denote nodal displacements and forces at nodes shared between the *k*th plate and the joint. Therefore, one can represent ${\mathbf {q}}_{LB,k}$ and ${\tilde{\mathbf{f}}}_{LB,k}$ in the wave mode shapes basis of the *k*th plate using Eq. (18) as follows

26 $$\begin{aligned} \begin{aligned} {\mathbf {q}}_{LB,k}&= {\mathbf {R}}_k \left( \mathbf {\Phi }^+_{{\mathbf {q}},k}{\mathbf {a}}^+_k +\mathbf {\Phi }^-_{{\mathbf {q}},k}{\mathbf {a}}^-_k\right) \\ {\tilde{\mathbf{f}}}_{LB,k}&= {\mathbf {R}}_k \left( \mathbf {\Phi }^+_{{\mathbf {f}},k}{\mathbf {a}}^+_k\, +\mathbf {\Phi }^-_{{\mathbf {f}},k}{\mathbf {a}}^-_k\,\right) \end{aligned} , \end{aligned}$$

where ${\mathbf {R}}_k$ is the matrix that transforms the local coordinate system of the *k*th plate to the global coordinate system. Since the local $x_k$ axis is aligned with the global $x_J$ axis, the transformation matrix ${\mathbf {R}}_k$ can be written as

27 $$\begin{aligned} {\mathbf {R}}_k = \begin{bmatrix} {\mathbf {R}}_{\text {node}} &{} 0 &{} \cdots &{} 0 \\ 0 &{} {\mathbf {R}}_{\text {node}} &{} \cdots &{} 0 \\ \vdots &{} \vdots &{} \ddots &{} \vdots \\ 0 &{} 0 &{} \cdots &{} {\mathbf {R}}_{\text {node}} \end{bmatrix}_{m_k\times m_k} , \quad {\mathbf {R}}_{\text {node}} = \begin{bmatrix} 1 &{} 0 &{} 0 \\ 0 &{} \cos (\psi _k) &{} -\sin (\psi _k) \\ 0 &{} \sin (\psi _k) &{} \cos (\psi _k) \\ \end{bmatrix} \end{aligned}$$

where $\psi _k$ denotes the angle of rotation between $y_k$ and $y_J$. Now, we can concatenate individual expressions (26) for ${\mathbf {q}}_{LB,k}$ and ${\tilde{\mathbf{f}}}_{LB,k}$ to express ${\mathbf {q}}_{J,\text {red}}$ and ${\mathbf {f}}_{J,\text {red}}$ in Eq. (23) as

28 $$\begin{aligned} \begin{aligned} {\mathbf {q}}_{J,\text {red}} = {\mathbf {R}} \biggl (\mathbf {\Phi }^+_Q{\mathbf {A}}^+ + \mathbf {\Phi }^-_Q{\mathbf {A}}^-\biggl ) \\ {\mathbf {f}}_{J,\text {red}} = {\mathbf {R}} \biggl (\mathbf {\Phi }^+_F{\mathbf {A}}^+ + \mathbf {\Phi }^-_F{\mathbf {A}}^-\biggl ) \end{aligned} \end{aligned}$$

with

29 $$\begin{aligned} {\mathbf {R}} \!=\! \begin{bmatrix} {\mathbf {R}}_1 &{} \cdots &{} 0 \\ \vdots &{} \ddots &{} \vdots \\ 0 &{} \cdots &{} {\mathbf {R}}_N \end{bmatrix} , \mathbf {\Phi }^\pm _Q \!=\! \begin{bmatrix} \mathbf {\Phi }^\pm _{{\mathbf {q}},1} &{} \cdots &{} 0 \\ \vdots &{} \ddots &{} \vdots \\ 0 &{} \cdots &{} \mathbf {\Phi }^\pm _{{\mathbf {q}},N} \end{bmatrix} , \mathbf {\Phi }^\pm _F \!=\! \begin{bmatrix} \mathbf {\Phi }^\pm _{{\mathbf {f}},1} &{} \cdots &{} 0 \\ \vdots &{} \ddots &{} \vdots \\ 0 &{} \cdots &{} \mathbf {\Phi }^\pm _{{\mathbf {f}},N} \end{bmatrix} , \, {\mathbf {A}}^\pm \!=\! \begin{bmatrix} {\mathbf {a}}^\pm _1 \\ \vdots \\ {\mathbf {a}}^\pm _N \end{bmatrix} . \end{aligned}$$

Inserting Eq. (28) in Eq. (23) yields

30 $$\begin{aligned} {\mathbf {A}}^+ = {\mathbf {S}}\,{\mathbf {A}}^- , \quad \text {with} \quad {\mathbf {S}} = -\left( {\tilde{\mathbf{D}}}_J{\mathbf {R}}\mathbf {\Phi }^+_Q-{\mathbf {R}} \mathbf {\Phi }^+_F\right) ^{-1}\left( {\tilde{\mathbf{D}}}_J{\mathbf {R}} \mathbf {\Phi }^-_Q-{\mathbf {R}}\mathbf {\Phi }^-_F\right) . \end{aligned}$$

The expression for ${\mathbf {S}}$ defines a scattering matrix relating the amplitudes ${\mathbf {A}}^-$ and ${\mathbf {A}}^+$ of incoming and outgoing waves, respectively. The dimension of the square matrix ${\mathbf {S}}$ is $\sum _{k=1}^N m_k$, and the scattering coefficients have the form $s^{nm}_{ij}(\omega ,k_x)$, relating an incoming wave *i* in the plate *n* and a reflected or transmitted wave *j* in the plate *m* at angular frequency $\omega$ and wave number component $k_x$. For the associated energy fluxes, we obtain the energy scattering coefficients as

31 $$\begin{aligned} t^{nm}_{ij}\left( \omega ,k_x\right) = {\left\{ \begin{array}{ll} \displaystyle \frac{J^+_{j,m}}{J^-_{i,n}} |s^{nm}_{ij}|^2 &{} \text {if wave }j \text {is propagating}. \\ 0 &{} \text {otherwise}. \end{array}\right. } , \end{aligned}$$

where

32 $$\begin{aligned} {\left\{ \begin{array}{ll} J^-_{i,n} &{}= \big |{\text {Re}}\left( \mathrm {i}\omega \phi ^{-^*}_{q,i,n}\,\phi ^-_{f,i,n}\right) \big | . \\ J^+_{j,m} &{}= \big |{\text {Re}}\left( \mathrm {i}\omega \phi ^{+^*}_{q,j,m}\, \phi ^+_{f,j,m}\right) \big | . \end{array}\right. } \end{aligned}$$

In the absence of damping, total energy must be conserved, hence the sum of the energy scattering coefficients over the outgoing modes equals one, that is,

33 $$\begin{aligned} \sum \limits _{m=1}^N\sum \limits _{j} t^{nm}_{ij} = 1 . \end{aligned}$$

## Numerical case examples

In this section, we present two numerical case studies to show the applicability of the method discussed above. In the first example, we consider an L junction of composite symmetric cross-ply laminated plates, i.e. laminates that consist of an odd number of orthotropic layers with principal material directions alternating between $0^{\circ }$ and $90^{\circ }$ for cross-ply laminates. In the second example, an L-type junction of composite angle-ply laminated plates is considered. In this case, laminates consist of an odd number of orthotropic layers with principal material directions alternating between $\alpha ^{\circ }$ and $-\alpha ^{\circ }$, $\alpha \in (0^{\circ },90^{\circ })$.

In all cases, we compute the energy scattering coefficients with respect to the wave number component $k_x$ for a fixed frequency *f*. The results for cross- and angle-ply laminated plates are compared with those obtained using the semi-analytical approach based on the solution of wave equations in the line-junction approximation^20^. We assume that the system is undamped; however, to facilitate the wave tracking process described in “Incoming and outgoing waves and the wave basis”, a small damping coefficient $\eta = 0.00001$ is applied.

### Cross-ply laminated plates

**Figure 6:**
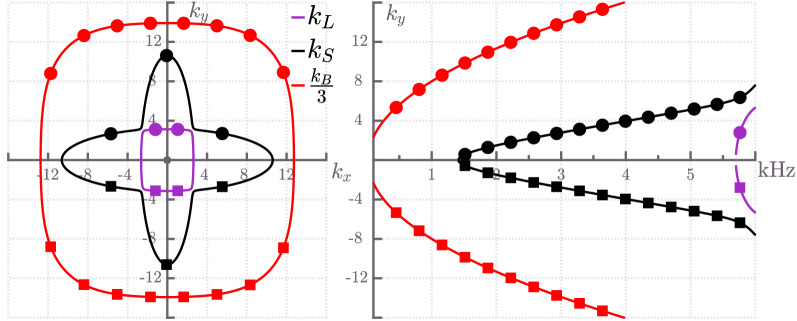
Wave vector (left) and dispersion (right) curves for a cross-ply composite plate. Longitudinal ($k_L$), shear ($k_S$) and bending ($k_B$) curves are depicted in purple, black and red colour, respectively. Squares and circles denote wave numbers of incoming and outgoing waves, respectively. The wave vector curves are plotted for a fixed frequency $f^0=3000$ Hz, whereas the dispersion curves are plotted for a fixed wave number component $k^0_x=5 \, \text {m}^{-1}$.

A five-layer symmetric cross-ply laminated plate of the total thickness of $h = 0.005$ m is considered. The material characteristics of all layers are the same and given in Table 1. The lamination scheme is $0^{\circ }/90^{\circ }/0^{\circ }/90^{\circ }/0^{\circ }$.

A periodic cell of length $d_x=0.001$ m and width $d_y=0.001$ m is modelled in ANSYS with 3 SOLID185 elements per ply, i.e. 15 finite solid elements in total. The usage of only one element in the cross-section is justified since the laminates considered are homogeneous in their plane dimensions. However, there must be at least 6–10 FE elements per wavelength to obtain accurate results. In other words, the wave numbers $k \le \frac{2\pi }{10\, max(d_x,d_y)}$ can be computed accurately. One can use more elements in the cross-section to alleviate round-off errors due to truncation of inertia terms in the dynamic stiffness matrix if needed^35^.

As presented in “Computation of wave numbers and eigenvectors”, solving (11) or (13) can yield propagating wave vector pairs $\left( k_x,k_y\right)$ for a fixed frequency $\omega ^0$ or dispersion curves $k_y = k_y(\omega ,k^0_x)$ for a fixed wave number component $k^0_x$. Figure 6 presents bending, shear and longitudinal wave vector curves for a fixed frequency $f^0=3000$ Hz on the left side and the correspondent dispersion curves for a fixed wave number component $k^0_x=5\, \text {m}^{-1}$ on the right side. These numerical dispersion relations can be used to calculate the group velocity angles and, therefore, propagation angles of transmitted waves via modified Snell’s law.

Next, we consider two identical cross-ply composite plates connected through an L-joint. The corresponding FE model is similar to the model shown in Fig. 5, but now only connecting $N=2$ plates. The FE model of the joint consists of 55 SOLID185 elements; thus, the dimensions of the joint stiffness and mass matrices are 164-by-164.

**Figure 7:**
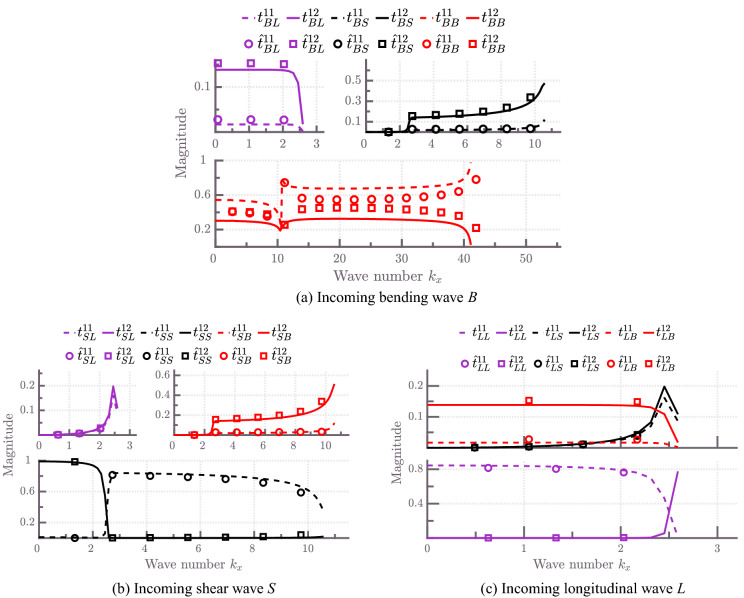
Energy scattering coefficients of an L-junction of two SOLID185-based cross-ply laminated plates for various incident waves plotted with respect to the wave number component $k_x$ at a frequency $f=3000$ Hz. ${\hat{t}}^{nm}_{ij}$ denote semi-analytical scattering coefficients obtained using the method presented in^20^.

Figure 7 presents a comparison of the energy scattering coefficients obtained from the current approach and the semi-analytical approach based on the work of Aimakov et al.^20^ for various incident modes as a function of wave number component $k_x$ at frequency $f=3000$ Hz. We note that the semi-analytical approach is based on further approximations, such as treating the junction as a one-dimensional line and is considered here only as a reference. Deviations between the WFE approach and the semi-analytical approach are mainly due to inaccuracies in the semi-analytical treatment. All solid and dashed lines represent numerical results, whereas circles and squares show semi-analytical results. Excellent agreement between numerical and semi-analytical energy scattering coefficients can be noted for incoming longitudinal and shear waves in Fig. 7b,c , respectively. Hence, modelling coupled thin composite plates using the semi-analytical method is sufficient for accurately estimating in-plane wave energy scattering coefficients.

For the case of an incoming bending wave, discrepancies between the WFE and the semi-analytical results appear. For instance, in Fig. 7a, it can be seen that semi-analytical results underestimate energy reflection and hence, overestimate energy transmission of an L-junction of plates. In fact, the maximum difference observed between bending using the WFE and semi-analytical results is $\sim$ 20 to 22%. This can be referred to the fact that the semi-analytical model results are based on the assumption that the joint can be represented as a shared one-dimensional line between plates^20^. This assumption breaks down at higher frequencies since the influence of the internal joint geometry becomes significant. Notably, the shear strain becomes more critical in the dynamic response of the joint and this effect is not considered in the semi-analytical model^49,66^.

The energy scattering coefficients of coupled shear-bending waves are equal, that is, $t^{11(12)}_{SB} = t^{11(12)}_{BS}$. The same applies for shear-longitudinal wave coupling, that is, $t^{11(12)}_{SL} = t^{11(12)}_{LS}$. The summation to unity of the energy reflection and transmission coefficients validates the numerical results obtained.

### Angle-ply composite plates

A five-layer angle-ply laminated plate of the total thickness of $h = 0.005$ m is considered. The material characteristics of the individual layers are the same as in the previous example. The lamination scheme is $45^{\circ }/-45^{\circ }/45^{\circ }/-45^{\circ }/45^{\circ }$. A periodic cell of length $d_x=0.001$ m and width $d_y=0.001$ m is again modelled in ANSYS with 15 SOLID185 elements.

**Figure 8:**
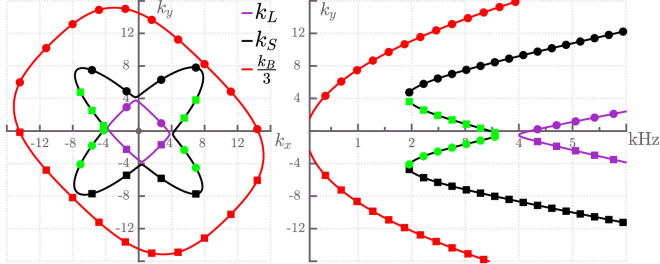
Wave vector (left) and dispersion (right) curves for an angle-ply composite plate. Longitudinal ($k_L$), shear ($k_S$) and bending ($k_B$) curves are depicted in purple, black and red colour, respectively. Squares and circles denote wave numbers of incoming and outgoing waves, respectively. Furthermore, green squares and circles represent parts of the shear wave vector and dispersion curves that exhibit negative group velocity behaviour. The wave vector curves are plotted for a fixed frequency $f=3000$ Hz, whereas the dispersion curves are plotted for a fixed wave number component $k_x=5 \, \text {m}^{-1}$.

Figure 8 presents bending, shear and longitudinal wave vector curves of an angle-ply laminated plate for a fixed frequency $f=3000$ Hz on the left side and the correspondent dispersion curves for a fixed wave number component $k_x=5\, \text {m}^{-1}$ on the right side. Note that there are two shear wave dispersion curves present on both sides of Fig. 8. The second shear wave (plotted in green) exhibit a negative group velocity phenomenon—they will be denoted as $S_2$ from now on. Details about these features in an orthotropic plate are discussed in^20^.

**Figure 9:**
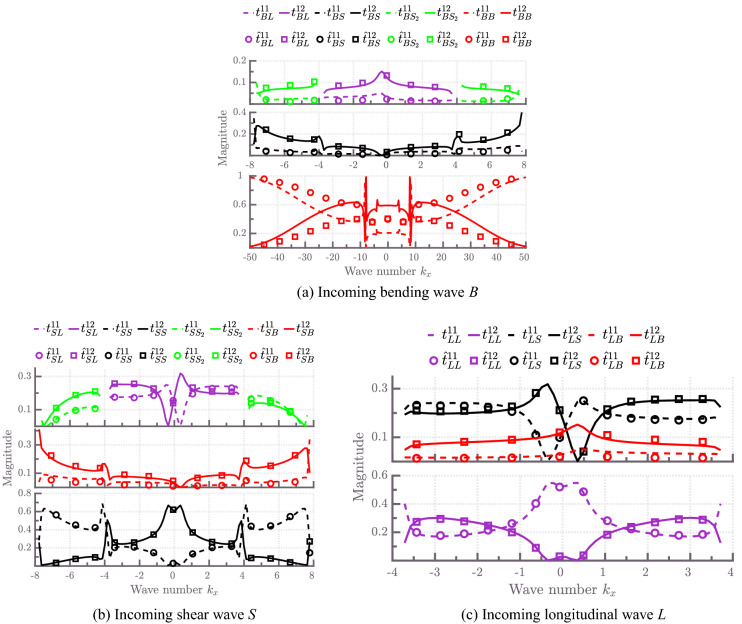
Energy scattering coefficients of an L-junction of two SOLID185-based angle-ply laminated plates for various incident waves plotted with respect to the wave number component $k_y$ at a frequency $f=3000$ Hz. ${\hat{t}}^{nm}_{ij}$ denote semi-analytical scattering coefficients obtained using the method presented in^20^.

A comparison of energy scattering coefficients obtained from the current approach and from the semi-analytical method is given in Fig. 9 for various incident modes as a function of wave number component $k_x$ at frequency $f=3000$ Hz. Again, numerical and semi-analytical energy scattering coefficients agree well for incoming longitudinal and shear modes, see Fig. 9b,c .

Deviations in the shape of energy reflection and transmission coefficients can be seen for an incoming bending wave in the case of SOLID185-based numerical results, see Fig. 9a. This phenomenon can be explained by the fact that the semi-analytical approach produces effective one-layer plates joined along the shared edge, thus losing the complexity of the connection between individual layers of plates at the junction.

As in the case of cross-ply laminated plates, curves describing the coupling between shear and bending waves are symmetric for a range of wave number components $|k_x| < 7.6\, \text {m}^{-1}$—the correspondent scattering coefficients obey $t^{11(12)}_{SB}=t^{11(12)}_{BS}$. Similarly, the scattering coefficients of longitudinal and bending coupled waves $t^{11(12)}_{LB}$ and $t^{11(12)}_{BL}$ are equal for $|k_x| < 3.7\, \text {m}^{-1}$. Furthermore, energy scattering coefficients of longitudinal and shear coupled waves are symmetric around $k_x=0\, \text {m}^{-1}$, that is, $t^{11(12)}_{SL}(k_x) = t^{11(12)}_{LS}(-k_x)$.

## Conclusion

A hybrid FE/WFE model has been developed predicting the scattering properties for different junctions of two-dimensional anisotropic composite plates. The influence of the angle of incidence on the distribution of the power flow of incident bending, shear and longitudinal type waves has been investigated. Numerical results presented were compared with semi-analytical evaluations of scattering coefficients. The method gives for the first time a detailed recipe for computing scattering coefficients for the generic case of an arbitrary number of composite plates connected at a junction without restrictions on the angles at which the plate meet or the orientation of the principal axes of individual plates. The results of this paper can be used for the computation of wave energy distributions in SEA by providing coupling loss factors^67^ and for angle-of-incidence dependent scattering coefficients entering the radiative transfer and DEA method.
